# Prevalence of impacted and supernumerary teeth among young Peruvians: A large cross-sectional study

**DOI:** 10.4317/jced.63746

**Published:** 2026-02-26

**Authors:** Lilian Damián-Navarro, Manuel Esquivel-Ortega, Jhoana Mercedes Llaguno-Rubio, Gustavo Adolf Fiori-Chincaro, Luis Ernesto Arriola-Guillén

**Affiliations:** 1 Division of Oral and Maxillofacial Radiology, Faculty of Dentistry, Centro Universitário do Norte de São Paulo (UNORTE), São Paulo, Brazil; 2 Division of Oral Radiology, Instituto Latinoamericano de Altos Estudios en Estomatología (ILAE), Lima, Peru; 3 Division of Orthodontics, Faculty of Dentistry, Universidad Científica del Sur, Lima, Peru

## Abstract

**Background:**

To determine the prevalence of impacted teeth (IT) and supernumerary teeth (ST) in young Peruvian individuals aged 13 to 20 years based on panoramic radiographs taken between 2020 and 2025. Materials and

**Material and Methods:**

This cross-sectional and retrospective study complied with all ethical standards. Digital panoramic radiographs from two radiological centers in Peru (n = 7,903) were evaluated to analyze and detect the presence of impacted and supernumerary teeth. All DPRs were assessed by two previously trained and calibrated investigators. Chi-square tests and binary logistic regression analyses were used, with a significance level set at p &lt; 0.05.

**Results:**

The study found a prevalence of impacted teeth (IT) of 58.7%, with no significant difference between sexes (p = 0.361). The prevalence of supernumerary teeth (ST) was 3.9% (males 5.5%, females 2.5%; p &lt; 0.001). Single ST were observed in 68.9% of cases, with no sex-related differences (p = 0.195). No maxillary or mandibular predominance was found (p = 0.717); however, a higher frequency of ST was recorded in the posterior region (56.1%, p = 0.088) and of the supplemental type (51.9%), with no differences by sex (p = 0.185). Binary logistic regression analysis revealed a higher risk of ST in males (B = 2.28, p &lt; 0.001), with no influence of age (p = 0.200). The most frequently impacted teeth were third molars (n = 3,579), followed by canines (n = 1,101) and incisors (n = 60).

**Conclusions:**

The prevalence of impacted teeth in young Peruvian patients was within the high range reported in the literature, with no significant differences by sex or age. Third molars were the most commonly impacted teeth, followed by canines. The prevalence of supernumerary teeth was within the average range reported in previous studies, being higher in males, predominantly single and located in the posterior region, with no age-related effect. Periodic radiographic evaluations during adolescence are recommended for early detection, as well as further research in young patients during the critical period of completion of permanent dental maturation.

## Introduction

Dental anomalies are alterations of epithelial and mesenchymal tissues that may affect the process of odontogenesis, resulting in isolated alterations or occurring as part of a syndrome (1). These dental anomalies may be congenital or developmental and can affect tooth number, size, shape, color, texture, eruption, position, and structure (1 , 2). It has been reported that approximately 5% of the population is born with some type of hereditary anomaly, and around 60% of these anomalies affect the teeth, jaws, or face (1 , 3). Impacted teeth (IT) are those that fail to fully erupt into the jaws after the normal eruption period (4 , 5). Their etiology is associated with local factors such as lack of space, retention of primary teeth, root dilaceration, trauma, ankylosis of primary teeth, inflammatory or pathological lesions, and gingival fibrosis, as well as systemic factors including malnutrition, anemia, rickets, vitamin D deficiency, endocrine disorders, and syndromes, among others. A possible genetic factor in their development has also been suggested (6). Regarding prevalence, studies report wide variability worldwide, with an incidence of impacted teeth ranging from 3% to 68.6%, and a higher frequency in females than in males (7 - 10). According to studies conducted to date, third molars are the most commonly impacted teeth, accounting for more than 95% of all impacted teeth, followed by canines, central incisors, and premolars. Maxillary canine impaction is approximately 20 times more frequent than mandibular canine impaction. These conditions constitute risk factors for occlusal problems and the development of various pathological lesions highlighting the need to investigate the prevalence of impaction according to tooth type (11 , 12). Supernumerary teeth (ST), or hyperdontia, represent a numerical dental anomaly characterized by an excess in tooth number and form. Their etiology is unknown but has been associated with hyperactivity of the epithelial cells of the dental lamina or with the division of the dental follicle (13). ST have a reported prevalence ranging from 0.1% to 3.8%, with mesiodens being the most common type, typically located in the midline of the maxilla. This type of tooth may appear in the primary dentition and is most frequently observed in the young permanent dentition. Sex distribution studies have reported a higher prevalence in males than in females, with a ratio of approximately 2:1. Diagnosis is usually made through radiographic findings (6 , 14 - 16). The presence of ST may also be associated with genetic syndromes such as cleidocranial dysplasia, Down syndrome, Ehlers-Danlos syndrome, Fabry-Anderson disease, and Gardner syndrome (17 , 18). ST are classified according to their location as mesiodens, incisor, premolar, paramolar, and distomolar. Based on morphology, they are divided into supplemental teeth, which resemble adjacent teeth in shape and size, and rudimentary teeth, which are less developed and may present as conical, tuberculate, or molariform types. They are further classified as impacted or erupted. Their prevalence ranges from 1% to 3%, with mesiodens being the most common type, accounting for approximately 45% to 47% of cases (6 , 14 , 19). Both impacted teeth (IT) and supernumerary teeth can cause numerous complications, ranging from inflammation, pain, swelling, bone loss, and root resorption of adjacent teeth to the formation of cysts or odontogenic tumors. These secondary effects may lead to occlusal problems and dental malformations, negatively affecting patients' psychological well-being due to facial and dental aesthetic concerns (1 , 3 , 20). Early diagnosis is crucial for planning effective treatment and improving clinical outcomes (1 , 3 , 13). The purpose of the present study is based on the need to update and expand epidemiological information on the prevalence and patterns of impacted and supernumerary teeth in young populations, as well as to analyze their relationship with demographic variables in order to provide updated data that contribute to more effective diagnosis and clinical management in young patients. Therefore, this study aims to determine the prevalence of impacted and supernumerary teeth in young Peruvians aged 13 to 20 years evaluated using digital panoramic radiographs between 2020 and 2025.

## Material and Methods

- Study design The present study employed a retrospective and cross-sectional design, and it was approved by Centro Universitário do Norte de São Paulo (UNORTE), São Paulo, Brazil, complied with all ethical standards. In accordance with the Declaration of Helsinki, patient data confidentiality was safeguarded to prevent any possibility of identification. This study consisted of the evaluation of digital panoramic radiographs of patients aged 13 to 20 years from two radiological centers in the city of Lima, taken between January 2022 and September 2025. - Sample Size The sample size was calculated using the formula for estimating proportions. A prior pilot study on impacted teeth yielded a prevalence of 56%. Based on this estimate, a new sample size calculation was performed using a 99% confidence level and a precision of 3%, resulting in a minimum required sample size of 1,817 panoramic radiographs. However, a total of 12,808 digital panoramic radiographs were obtained, of which 4,905 were excluded for not meeting the inclusion criteria. Consequently, the final sample evaluated consisted of 7,903 digital panoramic radiographs. - Selection criteria The inclusion criteria comprised digital panoramic radiographs (DPRs) of patients aged 13 to 20 years, of both sexes, taken between January 2022 and September 2025, with good resolution and image quality. DPRs from patients with infections or sequelae of trauma, syndromes affecting the jaws, previous jaw surgery, image superimposition, ongoing or previous orthodontic treatment, incomplete data records, or duplicate radiographs from the same patient were excluded. - Variables measurements In this study, the variables evaluated included sociodemographic factors such as sex (male, female) and age (13 to 20 years). Dental anomalies were defined as the presence or absence of IT and ST, as well as their type, quantity, and location, which were assessed using the classification proposed by Patil et al. (20). A tooth was considered impacted (IT) when its eruption pathway was obstructed by an adjacent tooth, bone, or soft tissue. Considering the average eruption time, teeth were classified as impacted when they remained within the jaw for a minimum of two years beyond the corresponding mean eruption age. Tooth position was determined using Winter's angulation classification (12). A supernumerary tooth (ST) is an additional tooth in the normal dental series, whether erupted or unerupted, and may resemble or differ from other teeth within the same group. Tooth type and form were determined according to the classification proposed by Davidson et al. (19). Morphology was classified into two main categories: supplemental, referring to teeth with normal size and shape, and rudimentary, which includes teeth with abnormal morphology, presenting differences in size and/or shape. The digital panoramic radiographs were authorized by both private radiological centers. At Center 1, DPRs were obtained using an Italian NewTom Giano HR unit, operating at 76 kV and 7 mAs, with an exposure time of 16.9 seconds. At Center 2, digital panoramic radiographs were acquired using a Planmeca ProMax unit (Helsinki, Finland), operating at 84 kV and 16 mA, with an exposure time of 16 seconds. All digital panoramic radiographs (DPRs) were evaluated by two previously trained and calibrated investigators under the supervision of two expert radiologists and one orthodontist. Image assessment was performed through direct and sequential observation over a three-month period in an environment with controlled lighting conditions. To minimize observer bias, each observer analyzed a maximum of 70 images per day. The evaluations were performed using two devices: an Asus laptop with an Intel Core i7 processor at 1.30 GHz (Windows 11 Home Single, 64-bit operating system) and a screen resolution of 1920 × 1080 pixels, and a MacBook Air (M1, 2020) with an Apple M1 chip, 8 GB of memory, and a 13.3-inch Retina LED-backlit IPS display with a resolution of 2560 × 1600 pixels. - Statistical Analysis Statistical analysis was performed using SPSS Statistics software (version 24.0; IBM, Armonk, NY). Descriptive analysis was conducted using frequencies and percentages with 95% confidence intervals. Associations between variables were evaluated using the Chi-square test and binary logistic regression, with a statistical significance level set at p &lt; 0.05.

## Results

The final sample consisted of 7,903 patients, of which 4,071 (51.5%) were male and 3,833 (48.5%) were female. These represent the baseline characteristics of the analyzed population, with no statistically significant differences in frequency according to sex or age (Table 1).

Table 1

Table 2 shows that the prevalence of impacted teeth was the highest among the variables analyzed, reaching 58.7%, with no significant association with sex (p = 0.361).

Table 2

In contrast, the prevalence of supernumerary teeth was 3.9%, with a higher incidence in males (5.5%) compared to females (2.5%) (p &lt; 0.001). Table 3 evaluates the number of supernumerary teeth by sex.

Table 3

In both sexes, when supernumerary teeth are present, they usually consist of a single tooth in 68.9% of cases, with no significant differences between sexes (p = 0.195). Additionally, the presence of a supernumerary tooth was not more frequent in the maxilla than in the mandible in either sex (p = 0.717). Table 4 shows that the location of supernumerary teeth was predominantly in the posterior region in both sexes (56.1%) (p = 0.088), and the type of supernumerary tooth did not show significant differences in frequency according to sex (p = 0.185), with supplementary teeth present in 51.9% of cases and rudimentary teeth in 48.1%.

Table 4

Table 5 illustrates the influence of sex and age on the occurrence of supernumerary teeth using binary logistic regression.

Table 5

No significant influence of age was observed (p = 0.200); however, males showed a higher risk of developing a supernumerary tooth (B = 2.28, p &lt; 0.001). Table 6 describes the number of impacted teeth by sex.

Table 6

In both sexes, a maximum of 8 impacted teeth per person was recorded (0.40%). Third molars were the most frequently impacted teeth (n = 3,579), followed by canines (n = 1,101), while incisors were the least frequently impacted (n = 60). Finally, Table 7 examines the influence of sex and age on the incidence of impacted teeth using binary logistic regression.

Table 7

No significant influence of sex was found (p = 0.431); however, age showed a significant effect, with the risk of developing an impacted tooth increasing with each additional year (B = 1.62, p &lt; 0.001).

## Discussion

The evaluation of the presence of impacted and supernumerary teeth remains a concern in dentistry due to its impact on preventive management and therapeutic decision, making in daily clinical practice. In the present study, 7,903 digital panoramic radiographs of patients aged between 13 and 20 years were analyzed. This age range is particularly relevant, as it corresponds to the period of permanent dental maturation, during which these anomalies have greater clinical impact. The prevalence of impacted teeth varies among different ethnic and racial groups (6). The prevalence of impacted teeth in our study (58.7%) was considerably higher than that reported by Chu et al. (2003), who evaluated 7,486 panoramic radiographs and found a prevalence of 28.3%, mainly concentrated in the 20-29 year (55.1%) and 17-19-year (33.5%) age groups (10). Similarly, Da Silva et al. (9) (2023) reported a prevalence of 38.7% in a Brazilian population, a figure that is also lower but methodologically more comparable. These differences may be attributed to population genetic variations, differences in the age groups analyzed, and inclusion criteria. Nevertheless, our prevalence of 58.7% lies at the upper end of the worldwide reported range (3%-68.6%), suggesting particular characteristics of the evaluated Peruvian population. One possible reason for the high prevalence of tooth impaction in the evaluated age group (13 to 20 years) is that this condition is overestimated in this demographic. However, this is also the age when individuals most frequently seek dental and orthodontic treatment, leading to the discovery of impacted teeth. Therefore, this age range is considered optimal for evaluating this condition. In contrast, older age groups may already have undergone intervention for impacted teeth, potentially leading to an underestimation of prevalence in those populations. Moreover, a recent systematic review with meta-analysis conducted by Pinto et al. (21) (2024) estimated a global prevalence of impacted third molars of approximately 37%, with marked geographic variability: higher figures were reported in Asia (43.1%) and lower in Europe (24.5%). This heterogeneity supports the multifactorial nature of dental impaction and the influence of ethnic and genetic factors, helping to contextualize the differences observed among countries and to place our findings within this spectrum. Regarding sex, our study showed a slightly higher frequency in females (59.2%) compared to males (58.2%), with no statistically significant difference (p = 0.361). This finding is consistent with studies that have also found no association between sex and dental impaction, although it contrasts with reports such as that of Ahmad et al. (22) (2022), who observed a higher prevalence in females in a young Pakistani population. With respect to the type of tooth affected, our study confirms that third molars represent the main site of impaction (77.03%), followed by canines (23.69%), premolars, molars, and, to a lesser extent, incisors. This pattern is consistent with that described by Alhajj et al. (11) (2024) in a Yemeni population, where third molars, especially mandibular ones were the most frequently impacted, followed by maxillary canines. Likewise, the findings of Fardi et al. (8) support the high frequency of canine impaction when third molars are not considered, a result similarly reflected in our sample, in which canines ranked second after third molars. A relevant finding is that, among the total cases with impacted teeth, 33.4% presented four impacted teeth simultaneously, suggesting a bilateral pattern of third molar impaction. Marghalani et al. (4) (2025), in their retrospective study of 2,199 radiographs, reported similar patterns, with mandibular third molars being the most frequently impacted, highlighting the importance of early radiographic diagnosis. On the other hand, canine impaction deserves special attention, as it represents the second most frequent anomaly in our study. Alamri et al. (7) (2020) reported that canines accounted for 58.6% of all impacted teeth when third molars were excluded from the evaluation in a Saudi population. In our study, canines represented approximately 24% of total impactions, with a predominance in the maxilla over the mandible, consistent with established knowledge that maxillary canine impaction is approximately 20 times more frequent than mandibular impaction. Logistic regression analysis showed that age is a significant predictor of dental impaction (B = 1.62, p &lt; 0.001), with the risk of impacted tooth development increasing with each additional year. This finding is particularly relevant, as the studied age group (13-20 years) corresponds to the critical period for the eruption of third molars and permanent canines. The absence of a significant association with sex (p = 0.431) suggests that, in the young Peruvian population, other factors, such as racial diversity, may carry greater weight than gender differences in determining dental impactions (6). Regarding the prevalence of supernumerary teeth, we found a rate of 3.9%, which falls within the range reported in the literature (0.1% to 3.8%). However, we identified a significant difference by sex, with a higher prevalence in males (5.5%) than in females (2.5%) (p &lt; 0.001), yielding a male-to-female ratio of approximately 2.2:1. This pattern is consistent with the international literature (6 , 14 , 15). Regarding morphology, we found an almost balanced distribution between supplementary teeth (51.9%) and rudimentary teeth (48.1%), with no significant differences by sex (p = 0.185). The relatively high proportion of supplementary teeth in our study could explain the higher frequency in the posterior region, as supernumerary premolars and molars typically present a supplementary morphology. The results of the present investigation partially coincide with those reported by Brinkmann et al. (23) (2020) in their study on supernumerary teeth in a Spanish population, where the predominant location was the posterior region (56.1%). In our study 68.9% of cases presented a single supernumerary tooth, 21.8% presented two teeth, and smaller percentages corresponded to multiple supernumerary teeth. This pattern of single-tooth presentation agrees with the literature, which establishes that approximately 76-86% of hyperdontia cases occur as a solitary tooth. Hajmohammadi et al. (13) (2021) reported similar distributions in an Iranian population, with multiple cases being less common and generally associated with genetic syndromes, which were excluded from our study. The high prevalence of impacted third molars recorded in our study (77.03% of all impacted teeth) suggests the need for periodic radiographic evaluation in adolescents to allow for early intervention. Likewise, the presence of supernumerary teeth, although less frequent, requires special attention due to their potential to cause malocclusion, delayed eruption of permanent teeth, and the formation of cystic lesions. Early diagnosis through digital panoramic radiography, as used in this study, allows timely intervention before the development of complications (6). The main strengths of this study include the large sample size (n = 7,903), which provides more precise prevalence estimates; the use of high-quality digital panoramic radiographs; evaluation by calibrated observers; and the specific focus on a young population (13-20 years), a critical age range for these anomalies. Moreover, our study has several limitations that should be noted. First, its retrospective design limits our ability to control for confounding clinical variables and to establish causal relationships. Additionally, the data were collected from only two radiology centers in Lima, which may restrict the generalizability of our findings to the wider Peruvian population. Although these centers serve a large patient base, their results might not accurately represent other populations. Furthermore, excluding patients undergoing orthodontic treatment could lead to an underestimation of the actual prevalence of these anomalies. We also relied exclusively on panoramic radiographs for our assessment. While these images are suitable for screening, they have well-documented diagnostic limitations. This indicates that future studies using three-dimensional imaging could yield more comprehensive insights into the issue.

## Conclusions

The prevalence of impacted teeth in the evaluated subjects was high (58.7%), with no significant differences between sexes, although age was confirmed as an important predictor by increasing the risk of impaction with each additional year. Third molars constituted the most frequently impacted tooth type, followed by canines and premolars, with a notable bilateral pattern reflected in the high proportion of patients who presented four impacted teeth simultaneously. Regarding supernumerary teeth, the prevalence was 3.9%, with a marked predominance in males, who showed a significantly higher risk of presenting this anomaly, while age had no significant effect. Most cases presented a single supernumerary tooth, with a balanced distribution between both arches and a slight tendency toward posterior localization, without statistically significant differences in location or morphology. These findings highlight the importance of establishing systematic radiographic evaluation protocols in adolescent and young populations, especially for the early detection of impacted third molars and for the particular surveillance of males with an increased risk of supernumerary teeth.

## Figures and Tables

**Table 1:** Initial characteristics of the sample.

Sex	n=7903 (100%)	Age	
		Mean	SD
Female	4070 (51.5%)	15.96	2.38
Male	3833 (48.5%)	15.91	2.29

p=0.357 Student’s T-test

**Table 2:** Prevalence of Impacted and Supernumerary Teeth in the Evaluated Sample According to Sex.

Sex		Impacted Teeth		Total	P
		Absent	Present		
Female	n	1662	2408	4070	0.361
	%	40.8	59.2	100	
Male	n	1604	2229	3833	
	%	41.8	58.2	100	
Total	n	3266	4637	7903	
	%	41.3	58.7	100	
Sex		Supernumerary Teeth		Total	P
		Absent	Present		
Female	n	3969	101	4070	<0.001*
	%	97.5	2.5	100	
Male	n	3623	210	3833	
	%	94.5	5.5	100	
Total	n	7591	311	7903	
	%	96.1	3.9	100	

Fisher’s Exact Test* Significant

**Table 3:** Prevalence of Impacted and Supernumerary Teeth in the Evaluated Sample According to Sex.

Sex		Number of supernumerary teeth								Total	p
		1	2	3	4	5	6	7	8		
Female	n	74	19	3	3	2	0	0	0	101	0.195
	%	73.3	18.8	3.0	3.0	2.0	0.0	0.0	0.0	100.0	
Male	n	141	49	15	1	1	1	2	1	211	
	%	66.8	23.2	7.1	0.5	0.5	0.5	0.9	0.5	100.0	
Total	n	215	68	18	4	3	1	2	1	312	
	%	68.9	21.8	5.8	1.3	1.0	0.3	0.6	0.3	100.0	

Chi-Square test

**Table 4:** Relationship between location, region, and type of supernumerary teeth in the evaluated sample according to sex.

Sex		Location		Total	P
		Maxilla	Mandible		
Female	n	49	52	101	0.717
	%	48.5	51.5	100	
Male	n	108	103	211	
	%	51.2	48.8	100	
Total	n	157	155	312	
	%	50.3	49.7	100	
Sex		Region		Total	P
		Anterior	Posterior		
Female	n	37	64	101	0.088
	%	36.6	63.4	100	
Male	n	100	111	211	
	%	47.4	52.6	100	
Total	n	137	175	312	
	%	43.9	56.1	100	
Sex		Type		Total	P
		Supplementary	Rudimentary		
Female	n	58	43	101	0.185
	%	57.4	42.6	100	
Male	n	104	107	211	
	%	49.3	50.7	100	
Total	n	162	150	312	
	%	51.9	48.1	100	

Fisher’s exact test

**Table 5:** Binary logistic regression analysis of supernumerary teeth in relation to predictor variables.

Predictor variables	p	Exp(B)	95% CI for B	
			Lower	Upper
Age	0.200	0.97	0.92	1.01
Female				
Male	<0.001*	2.28	1.78	2.89
Constant	<0.001*	0.04		

*Significant

**Table 6:** Number of impacted teeth in the evaluated sample according to sex and tooth type.

Sex		Number of impacted teeth								Total
		1	2	3	4	5	6	7	8	
Female	N	426	559	376	867	110	47	21	10	2416
	%	17.60	23.10	15.60	35.90	4.60	1.90	0.90	0.40	100
Male	n	439	595	352	686	94	43	12	9	2230
	%	19.70	26.70	15.80	30.80	4.20	1.90	0.50	0.40	100
Total	n	865	1154	728	1553	204	90	33	19	4646
	%	18.60	24.80	15.70	33.40	4.40	1.90	0.70	0.40	100
Sex		Number of impacted incisors								Total
		1	2	3						
Female	n	23	2	0						25
	%	92	8	0						100
Male	n	31	3	1						35
	%	88.57	8.57	2.86						100
Total	n	54	5	1						60
	%	90	8.33	1.67						100
Sex		Number of impacted canines								Total
		1	2	3	4					
Female	n	313	180	46	17					556
	%	56.3	32.4	8.3	3.1					100
Male	n	320	154	48	23					545
	%	58.7	28.3	8.8	4.2					100
Total	n	633	334	94	40					1101
	%	57.5	30.3	8.5	3.6					100
Sex		Number of impacted premolars								Total
		1	2	3	4	5	6	7		
Female	n	141	54	14	10	1	2	1		223
	%	63.2	24.2	6.3	4.5	0.4	0.9	0.4		100
Male	n	129	31	11	1	2	1	0		175
	%	73.7	17.7	6.3	0.6	1.1	0.6	0.0		100
Total	n	270	85	25	11	3	3	1		398
	%	67.8	21.4	6.3	2.8	0.8	0.8	0.3		100
Sex		Number of impacted first and second molars								Total
		1	2	3	4					
Female	n	88	40	6	3					137
	%	64.2	29.2	4.4	2.2					100
Male	n	94	36	6	6					142
	%	66.2	25.4	4.2	4.2					100
Total	n	182	76	12	9					279
	%	65.2	27.2	4.3	3.2					100
Sex		Number of impacted third molars								Total
		1	2	3	4	5				
Female	n	189	430	311	938	1				1869
	%	10.1	23.0	16.6	50.2	0.1				100
Male	n	193	509	289	717	2				1710
	%	11.3	29.8	16.9	41.9	0.1				100
Total	n	382	939	600	1655	3				3579
	%	10.7	26.2	16.8	46.2	0.1				100

6

**Table 7:** Binary logistic regression analysis of impacted teeth in relation to predictor variables.

Predictor variables	p	Exp(B)	95% CI for B	
			Lower	Upper
Age	<0.001*	1.62	1.58	1.66
Female				
Male	0.431	0.96	0.86	1.06
Constant	<0.001*	0.001		

*Significant

## Data Availability

The data supporting the findings of this study are available from the corresponding author upon reasonable request.
